# Transplant-Associated Thrombotic Microangiopathy in Pediatric Hematopoietic Cell Transplant Recipients: A Practical Approach to Diagnosis and Management

**DOI:** 10.3389/fped.2019.00133

**Published:** 2019-04-09

**Authors:** Christopher C. Dvorak, Christine Higham, Kristin A. Shimano

**Affiliations:** Division of Pediatric Allergy, Immunology, and Bone Marrow Transplant, Benioff Children's Hospital, University of California, San Francisco, San Francisco, CA, United States

**Keywords:** thrombotic microangiopathy, atypical hemolytic uremic syndrome, hematopoietic cell transplantation, endothelial injury, complement

## Abstract

Transplant-associated thrombotic microangiopathy (TA-TMA) is an endothelial damage syndrome that is increasingly identified as a complication of both autologous and allogeneic hematopoietic cell transplantation (HCT) in children. If not promptly diagnosed and treated, TA-TMA can lead to significant morbidity (e.g., permanent renal injury) or mortality. However, as the recognition of the early stages of TA-TMA may be difficult, we propose a TA-TMA “triad” of hypertension, thrombocytopenia (or platelet transfusion refractoriness), and elevated lactate dehydrogenase (LDH). While not diagnostic, this triad should prompt further evaluation for TA-TMA. There is increased understanding of the risk factors for the development of TA-TMA, including those which are inherent (e.g., race, genetics), transplant approach-related (e.g., second HCT, use of HLA-mismatched donors), and related to post-transplant events (e.g., receipt of calcineurin inhibitors, development of graft-vs. -host-disease, or certain infections). This understanding should lead to enhanced screening for TA-TMA signs and symptoms in high-risk patients. The pathophysiology of TA-TMA is complex, resulting from a cycle of activation of endothelial cells to produce a pro-coagulant state, along with activation of antigen-presenting cells and lymphocytes, as well as activation of the complement cascade and microthrombi formation. This has led to the formulation of a “Three-Hit Hypothesis” in which patients with either an underlying predisposition to complement activation or pre-existing endothelial injury (Hit 1) undergo a toxic conditioning regimen causing endothelial injury (Hit 2), and then additional insults are triggered by medications, alloreactivity, infections, and/or antibodies (Hit 3). Understanding this cycle of injury permits the development of a specific TA-TMA treatment algorithm designed to treat both the triggers and the drivers of the endothelial injury. Finally, several intriguing approaches to TA-TMA prophylaxis have been identified. Future work on the development of a single diagnostic test with high specificity and sensitivity, and the development of a robust risk-scoring system, will further improve the management of this serious post-transplant complication.

## Introduction

Hematopoietic cell transplantation (HCT) for pediatric patients has classically been limited by transplant-related mortality (TRM). It has long been recognized that certain patients undergoing HCT are at increased risk for the development of endothelial injury, notably in the sinusoids of the liver, which manifests as sinusoidal obstruction syndrome (SOS). Recently, there has been an increased awareness that endothelial injury outside of the liver sinusoids is also a common occurrence in pediatric HCT recipients. This injury manifests itself as a thrombotic microangiopathy (TMA), with the most common features being hemolytic anemia, thrombocytopenia, hypertension, and renal dysfunction.

As a robust single-diagnostic test does not yet exist, the incidence of transplant-associated (TA)-TMA is poorly defined, but likely ranges from 10 to 35% (1). This variability is in large part due to the lack of standardly-applied consensus criteria as well as the significant limitations of the criteria themselves. Currently, the best criteria for pediatric patients are those proposed by Jodele et al. (2), while in our experience other criteria are less useful due to their reliance upon the presence of schistocytes (3–5). Despite issues with non-standardized definitions, one thing is certain: patients who develop TA-TMA have significantly higher rates of TRM (2, 6, 7), making the diagnosis and management of this disease of critical importance.

Globally, TA-TMA fits into the general classification of atypical hemolytic uremic syndromes (aHUS), as complement has shown to play a major role in the pathophysiology of the disorder. While post-HCT thrombotic thrombocytopenic purpura (TTP) has been reported (8), it is extremely uncommon compared to TA-TMA. Nevertheless, a first step toward treating a patient with suspected TA-TMA features is to rule out TTP by documenting normal levels (>10%) of ADAMTS13 and normal von Willebrand Factor (vWF) multimers, since treatment of TTP differs significantly from TA-TMA. Similarly, because the treatment is quite different from TA-TMA, ruling out classic HUS due to Shiga toxin-producing E. coli (STEC) is warranted, though it is also very rare to see that disease in post-HCT patient. However, onset of TMA symptoms 5–10 days following an episode of abdominal pain, vomiting, and diarrhea should prompt consideration of a stool culture and testing for the Shiga toxin. Finally, while a modified version of the Ham test has been shown to distinguish aHUS of transplant from other TMAs (9), in our experience this is generally not needed in clinical practice.

## Pathophysiology of TA-TMA

The pathophysiology of the vascular endothelial injury found in patients with TA-TMA is extremely complex, but can be simplistically broken down into three major pathways:

1. *Activation of endothelial cells to produce a pro-coagulant state*. When damaged by conditioning agents and other medications, activated endothelial cells undergo multiple changes:
  - Tissue Factor is expressed on the cell surface. This binds Factor VIIa (FVIIa), which promotes thrombus formation with activated platelets. Increased production of plasminogen activator inhibitor-1 (PAI-1) prevents fibrinolysis of the clot.
  - Decreased levels of NO and prostacyclin (PGI2) leads to loss of suppression of the activation of the coagulation cascade and complement. Hemolysis leads to production of free hemoglobin, which further decreases NO levels.
  - Decreased VEGF levels, in combination with release of ANG2 from endothelial cells and binding to the Tie2 receptor leads to vascular destabilization and vascular leakage.
  - Increased expression of adhesion molecules (E-selectin, ICAM-1, VCAM-1) permits the local recruitment of antigen presenting cells (APCs) and lymphocytes.
  - Damaged endothelial cells release IL-8.
  - Endothelial cells and cell-derived micro-particles are formed and released into the blood (10, 11).
2. *Activation of antigen-presenting cells (macrophages and neutrophils) and lymphocytes*. The activated endothelial cells in turn cause activation of a number of immune effector cells, including:
  - IL-8 activates neutrophils to release NETs (which activate complement via complement factor P/properdin) (12).
  - APCs express TNF-alpha, IFN-gamma, IL-1, IL-6, IL-8, and IL-12, further stimulating activation of T-cells and monocytes/macrophages.
  - IFN-gamma and TNF-alpha enhance class II HLA-antigen expression on endothelial cells, making them more susceptible to T-cell mediated alloreactivity.
  - Activated B-cells can potentially produce pathogenic antibodies. These include recipient-specific antibodies directed against class II HLA molecules (which can activate the classical complement cascade) (13), or anti-factor H auto-antibody production (which then prevent inhibition of the alternative complement pathway).
3. *Activation of the complement cascade and microthrombi formation*. The immunologic response activates both the alternative and classical complement cascades, as well as precipitates the formation of micro-thrombi:
  - Activation of C3 by hydrolysis leads to formation of C3b which covalently binds to an endothelial cell surface. C3b binding is further propagated by endothelial injury.
  - Once C3b is bound to the cell surface, complement Factor B (CFB) binds to C3b and is cleaved by complement factor D (CFD) leading to further amplification of alternative complement pathway, ultimately leading to C5 cleavage to C5a and C5b and formation of the lytic membrane attack complex (MAC, C5b-9) on the endothelial cell surface, causing direct endothelial cell lysis.
  - C5a, a potent anaphylatoxin, releases the anticoagulant molecule heparan sulfate from the surface of the endothelial cells.
  - Classical complement activation by antigen/antibody complexes actives C1 to trigger conversion of C4 to C4d, which remains covalently bound to tissues such as the glomeruli and intestines (14, 15).
  - Tissue damage and inflammation leads to increased endothelial permeability, red cell (RBC) and platelet extravasation, fibrin deposition, and micro-thrombi formation along microvascular wall resulting in affected organ ischemia.

The end sum of these pathways is the death of endothelial cells and micro-thrombi formation. An excellent illustration of the pathophysiology of TA-TMA can be found in Jodele et al. (1).

## Clinical and Laboratory Features of TA-TMA

In pediatric patients, TA-TMA typically occurs early post-allogeneic HCT, with a median diagnosis at 35–47 days post-HCT, and 88–92% occurring before Day +100 (2, 16). However, cases have been reported up to 2 years post-HCT (6, 17). Autologous recipients may develop TA-TMA even earlier, with a median of 18 days post-HCT (18). Not all features appear simultaneously, and in retrospect, it is often easy to appreciate that sub-clinical TA-TMA was developing for days, if not weeks, prior to the official diagnosis. As detailed below and in Table 1, prompt diagnosis of TA-TMA requires regular monitoring and trending of certain lab tests, especially in high-risk patients.

**Table 1:** Clinical and laboratory features of TA-TMA: differential diagnosis and evaluations.

**Clinical sign (in typical order of presentation)**	**Clues/tips**	**Differential diagnoses**	**Useful evaluations**
**THE TA-TMA “TRIAD” (PRESENT IN ALMOST ALL CASES)**			
**Hypertension (>95% for age, sex, height)**	Requiring >1 BP Med (or >0 if not on a CNI/steroids)	CNI, Steroids	
***de novo*** **Thrombocytopenia**/platelet transfusion refractoriness	No rise in platelet count the day after transfusion	SOS, Anti-platelet antibodies	Bilirubin, liver US Post-transfusion count Immature platelet fraction
**Elevated LDH** (>ULN for age)	Need to monitor twice weekly & trend	Liver injury, AIHA	Liver enzymes Direct antiglobulin test
**OTHER FINDINGS**			
**Proteinuria** **(≥30 mg/dL)**	Need to monitor UA daily Send Urine Protein/Cr if UA positive	Cystitis	Urine and blood PCR for BK virus & adenovirus
Elevated D-dimers	Need to monitor twice weekly and trend	Sepsis/DIC, SOS	Blood cultures
Falling haptoglobin	May rise first as an inflammatory acute phase reactant, such that the fall can be late	AIHA	Direct antiglobulin test
**New Anemia**/increased RBC transfusion needs		AIHA	Reticulocyte count
Rising creatinine	Late finding	CNI, Other meds (anti-virals), BK nephritis	Blood PCR for BK virus
**Schistocytes**	Often absent	Fairly pathognomonic, when present	

*Bold, Part of Current Diagnostic Criteria of Jodele et al. (2) along with sC5b9, though not all required to be present in order to make the diagnosis. BP, blood pressure; CNI, calcineurin inhibitor; SOS, sinusoidal obstruction syndrome; US, ultrasound; LDH, lactate dehydrogenase; ULN, upper limit of normal; AIHA, autoimmune hemolytic anemia; UA, urinalysis; DIC, disseminated intravascular coagulation; RBC, red blood cell*.

Hypertension is often the first clinical sign of impending TA-TMA, typically proceeding formal diagnosis by a week or more (2, 19). Unfortunately, hypertension is very common post-HCT, especially in patients receiving calcineurin inhibitors (CNI), such as tacrolimus and cyclosporine. A rule of thumb in the pediatric HCT recipient is to suspect TA-TMA whenever a patient requires one additional anti-hypertensive medications beyond what is typical for the patient's clinical situation (1). For example, it may be normal for a patient on a CNI to require one anti-hypertensive, but addition of a second agent should prompt an investigation for TA-TMA. Similarly, unless they are admitted with a history of hypertension, autologous recipients or patients receiving *ex vivo* T-cell depleted HCTs should not require any routine anti-hypertensive medications, and initiation of one should trigger consideration of impending TA-TMA.

The other clinical features, including thrombocytopenia [especially platelet transfusion refractoriness, as also seen in SOS (20)], elevated lactate dehydrogenase (LDH), proteinuria, anemia, and falling haptoglobin begin to appear shortly thereafter (2). All institutions should strongly consider routine monitoring of LDH twice weekly for the first 100 days post-HCT. In patients with thrombocytopenia and anemia, a critical distinction to make is hypoproduction (as part of the normal post-HCT marrow recovery or from marrow suppressive medications) vs. destruction (as in TA-TMA). A frequent interval between transfusions can be a clue to destruction, as can testing for elevations in the immature platelet fraction and the reticulocyte count, though this can be low early post-HCT. As the typical first three signs to manifest, hypertension, thrombocytopenia, and elevated LDH can be considered the “*TA-TMA triad*,” akin to the SOS classic triad of hepatomegaly, weight gain, and elevated bilirubin.

One of the diagnostic criteria suggests that patients with TA-TMA should have normal coagulation studies (prothrombin time and activated partial thromboplastin time) to exclude cases of disseminated intravascular coagulation (DIC) (3). However, in our experience this should not be considered a hard and fast rule, since patients may have other reasons for abnormal coagulation studies (such as prior or concomitant SOS). Furthermore, it is typically easy clinically to distinguish patients with DIC (who are usually tachycardic and hypotensive) from those with TA-TMA (who are invariably hypertensive). Fibrin D-dimers, a sign of intravascular clot formation and breakdown, are not part of any current criteria. However, in our experience they are extremely sensitive (albeit non-specific) marker for active TA-TMA, such that their absence should prompt consideration of alternative causes for the patient's clinical signs and symptoms. One report suggested that D-dimers, in combination with other markers of endothelial injury (angiopoietin-2 [ANG2], C-reactive protein [CRP], and thrombomodulin [TM]), can help define TA-TMA as well as the risk of subsequent TRM (21). Others have also noted increased D-dimer levels in TA-TMA (22).

Although not part of the current criteria, in addition to proteinuria, hemoglobinuria is also commonly present. Rising creatinine can be a late feature of TA-TMA and is fairly concerning that irreversible kidney injury has begun. Cystatin C may be a better indicator of early renal dysfunction, but it is less readily available (23). With the other diagnostic features present and other diagnoses on the differential list excluded, we firmly believe that clinicians should not wait for signs of renal injury in order to diagnose TA-TMA. A significant percentage of patients eventually go on to develop renal failure requiring the initiation of dialysis (6).

The presence of schistocytes on a peripheral blood smear should be the pathognomonic marker of TA-TMA, but because this is a subjective test requiring interpretation from a skilled microscopist, at many institutions it may be of very limited utility and therefore should not be relied upon to verify the diagnosis of TA-TMA (24). There is also the possibility that high vascular permeability leads to extravasation of the fragmented erythrocytes so that they are no longer in active circulation (19).

When all else fails, histologic evidence of microangiopathy on biopsy of an affected organ (e.g., kidney or intestine) is the gold standard to the diagnosis of TA-TMA. In our experience, however, such invasive procedures are rarely required to confirm the diagnosis and, in patients with severe thrombocytopenia from TA-TMA, are associated with a significant risk of serious bleeding.

While the hallmark of TA-TMA is kidney involvement, it is important to recognize that other organs may also be compromised in TA-TMA, especially the gastrointestinal tract (manifested as abdominal pain, nausea, bleeding, and ileus) (25), serosal surfaces (manifested as pericardial and pleural effusions) (26, 27), the cardiopulmonary system (manifested as pulmonary arterial hypertension) (28), and the neurologic system (manifested as confusion, headaches, and seizures) (1).

## Investigations at First Clinical Suspicion of TA-TMA

1. Ruling Out Other Diagnoses on the Differential
  - Other TMAs: As noted above, documentation of a normal ADAMTS13 level and negative stool Shiga toxin are technically warranted.
  - Autoimmune hemolytic anemia (AIHA): Direct antibody testing (Coombs) should be performed on all post-HCT patients with anemia, elevated LDH, and low haptoglobin. In rare cases, patients may have both AIHA and immune thrombocytopenia (ITP) post-HCT, but even this is typically distinguishable from TA-TMA due to the lack of hypertension and proteinuria in the former setting.
  - Liver SOS: TA-TMA shares certain features with SOS, especially the platelet refractoriness and fluid retention. However, coagulation studies (such as the INR and aPTT) are typically normal in TA-TMA and elevated in SOS. Bilirubin may not always be elevated in pediatric patients with SOS, so a liver ultrasound with Doppler evaluation for reversal of portal venous flow may be useful (20).
  Overall, few diseases in HCT patients fully mimic the entire picture of TA-TMA and therefore, in at-risk patients (see below), the development of associated signs and symptoms should be considered TA-TMA until proven otherwise. In other words, when you hear the TA-TMA triad of “hypertension, thrombocytopenia, and elevated LDH,” TA-TMA should be considered a “horse,” not a “zebra.”
2. *Evaluating the Extent and Risk of TA-TMA* (Table 2)
  - Complement system: C3 levels may be low in patients with TA-TMA, suggesting a state of complement activation (29). Additionally, serum complement membrane attack complex sC5b9 levels may be elevated due to terminal complement activation. Although a supportive feature for the diagnosis, not all patients with TA-TMA have an elevated sC5b9 level. Patients with elevated sC5b9 levels, however, are at increased risk of death from TA-TMA (2), and so this test can serve as a marker for patients who absolutely should be treated with complement blockade (see below). When complement blockade is utilized as treatment, CH50 levels are a marker of efficacy, and a baseline CH50 therefore should be performed as part of the workup.
  - Gastrointestinal tract: The gastrointestinal (GI) tract is often involved with TA-TMA and can present with symptoms which mimic GI GVHD, such as diarrhea, vomiting, pain, and bleeding (25). If the patient does not have evidence of GVHD elsewhere, an endoscopy with biopsies may be required to distinguish incompletely-treated TA-TMA vs. active GVHD, though the treatments for these may overlap (see below).
  - Serosal surfaces: While classically thought of as a manifestation of chronic graft- vs. -host disease (GVHD), in our experience all patients with evidence of pleural and pericardial effusions on chest radiograph and echocardiogram should be evaluated for TA-TMA (26). If clinically significant (i.e., respiratory distress or tamponade), the fluid may require drainage. However, because the fluid will continue to re-accumulate until the pathophysiology behind the serositis resolves, a one-time drainage procedure, such as pleurocentesis or pericardiocentesis is typically insufficient. If an intervention is to be performed, drains should typically be left in place until there are signs that the serositis is improving. For patients with pericardial effusions, in order to minimize the infectious risk of a prolonged external drain, a pericardial window may be necessary.
  - Cardio-pulmonary: Cardiac evaluation should be performed on all patients with TA-TMA, as they are at increased risk for pulmonary arterial hypertension (PAH), which if not recognized and treated, may be deadly (1, 28). Clinically, hypoxemia or respiratory distress may be noted, but as these are late findings, work-up should proceed even in asymptomatic patients (1). An electrocardiogram may demonstrate right axis deviation, while the echocardiogram needs to be requested to specifically evaluate for evidence of right ventricular (RV) dysfunction, such as a RV pressure >35% (50% in adults) of systemic and a tricuspid regurgitation velocity of >2.9 m/second (1). Any evidence of PAH should prompt evaluation by an experienced PAH-team who can assist with further work-up and management. One report noted that an echocardiogram done on all patients at Day +7 post-HCT may be an excellent tool for screening which patients are about to develop clinically-apparent TA-TMA (27).
  - Neurologic: Tight control of hypertension can potentially mitigate neurologic complications, but if such symptoms develop, a brain MRI should be performed. It may demonstrate bilateral white matter abnormalities in the vascular watershed areas consistent with posterior reversible encephalopathy syndrome (PRES) (30).
  - Immunologic evaluation: Evaluating and trending a CRP can be a useful marker of excessive inflammation (21). While most TA-TMA is mediated by complement, rare patients can instead have a process driven by autoantibodies, typically to Complement Factor H (CFH), a negative regulator of the alternative pathway (31). It is also thought that other recipient-specific autoantibodies against class II HLA-molecules or other endothelial proteins may be involved (13, 32). One potential clue to this rare occurrence may be a sudden rise in B cell numbers on lymphocyte phenotyping or other autoimmune manifestations. An ELISA test for anti-CFH antibodies is also clinically available.
  - Genetics: Primary atypical HUS occurring outside of the setting of HCT is typically mediated by gain-of-function mutations in genes encoding complement or by deficiencies in genes responsible for complement regulatory proteins (33). These pathologic mutations drive microangiopathy without significant stressors, but are very rare (<five per million). Conversely, 65% of TA-TMA patients will have one or more “benign” variants in complement genes detected. Although not urgent, testing for mutations in complement genes may be useful when determining length of complement blockade therapy, if used (see below). Commercial tests for mutations in CFH, MCP (CD46), CFI, C3, CFB, CFHR1, CFHR3, CFHR4, CFHR5, TM, Plasminogen, and DGKE are all available. Obviously, because these are host proteins, in the allogeneic setting this test must be done with DNA obtained from a buccal swab or pre-HCT banked DNA.

**Table 2:** Evaluations to perform after confirmation of TA-TMA.

**Evaluation**	**Notes**
Complement: C3, sC5b-9, CH50	C3 level is typically low (representing consumption) Elevated sC5b9 is a risk-factor for poor outcome Baseline CH50 useful if eculizumab is to be administered
Reticulocyte count (trend) and Immature Platelet Fraction (trend)	Useful to determine if marrow production is able to keep up with destruction
GVHD biomarkers: ST2, REG3-alpha, ± HGF, ± Elafin	Useful to determine if GVHD is driving the TA-TMA
Chimerism (T-cell)	May be useful to determine if sub-clinical GVHD is driving the TA-TMA
CRP (trend)	May be useful to determine if uncontrolled inflammation is driving the TA-TMA
Viral PCRs (CMV, Adenovirus, HHV-6, BK) and Galactomannan	Useful to determine if uncontrolled infection is driving the TA-TMA
Antibodies to CFH & Class II HLA	Marker of antibody-mediated TA-TMA
Electrocardiogram and Echocardiogram	Request specific evaluation of RV pressure and Tricuspid Regurgitation Velocity
Consider genetic panel (complement)	Must be done on buccal swab or banked DNA (if allogeneic HCT)

*GVHD, graft-vs.-host disease; ST2, suppressor of tumorigenicity 2; REG3, regenerating islet-derived 3; HGF, hepatocyte growth factor; CRP, C-reactive protein; CMV, cytomegalovirus; HHV-6, human herpes virus-6; CFH, complement factor H; HLA, human leukocyte antigen*.

## Risk Factors

Especially in the allogeneic setting, numerous risk factors have been identified. These can be separated into those that are: (1) inherent and non-modifiable; (2) transplant approach-associated; and (3) post-transplant event related (Table 3).

1. Inherent/Non-Modifiable Risk Factors

**Table 3:** Potential risk factors for TA-TMA.

**Inherent/non-modifiable**	**Transplant-associated**	**Post-transplant events**
Female sex	HLA-mismatched donor	CNI ± Sirolimus
Gene variants	Minor ABO mismatch	Infection (Bacteremia or IFI)
African-American race	Use of PBSC	Infection (DS-DNA Virus, e.g., CMV, HHV-6, BK)
Severe aplastic anemia	Lack of ATG in conditioning regimen	Acute GVHD
CMV seropositive recipient	Myeloablative conditioning	Autoantibody formation (Factor H, others)
Prior HCT	Slow metabolism of conditioning agents?	

*HLA, human leukocyte antigen; CNI, calcineurin inhibitor; ABO, blood type antigens; IFI, invasive fungal infection; PBSC, peripheral blood stem cells; GVHD, graft-vs. -host disease; CMV, cytomegalovirus; HHV-6, human herpes virus-6; ATG, anti-thymocyte globulin; HCT, hematopoietic cell transplant*.

Genetics may play an important role in determining who eventually develops TMA following an HCT. Variants in complement genes are commonly found in all races, but when multiple variants are found in an individual, the risk of subsequent TA-TMA is significantly elevated (34). African-American patients have been reported to have higher rates of TA-TMA, perhaps because of an increased likelihood of possessing more than one of the aforementioned activating variants in complement genes due to a protective effect against Neisseria infections (34). These “benign” gene variants are considered insufficient to trigger aHUS in the absence of a serious endothelial stressor; however, they become clinically apparent in the post-transplant period. As most complement is produced in the liver, these gene variants are typically host in nature, although one report suggested that donor mutations may also be responsible, since some complement appears to be made in monocytes and platelets (35).

Another report suggested that variants in CD40L, a gene responsible for production of a protein which plays a central role in co-stimulation and regulation of the immune response via T cell priming and activation of CD40-expressing immune cells, may increase the risk of TA-TMA (36). Finally, one report suggested that carriers of HLA-DRB11 are at increased risk of developing TA-TMA (37).

Surprisingly, females have been noted to have an increased incidence compared to males in several reports (6, 16, 38), though the pathophysiologic basis for this finding is poorly understood. One study of adults also noted an increased risk of TMA in CMV seropositive recipients (39).

Having severe aplastic anemia (SAA) as an underlying primary disease has been linked to increased rates of TA-TMA (2). One hypothesis is that this is related to prolonged exposure to CNIs pre-transplant. If so, differential rates should be seen in those patients with SAA who proceed straight to HCT vs. those who undergo immunosuppressive treatment first, but to date this analysis has not been performed. Patients with chronic hemolytic conditions such as sickle cell disease or beta-thalassemia may also be at elevated risk, though the data for this is currently limited (30).

2. *Transplant Approach-Associated Risk Factors*

In allogeneic HCT recipients, a prior transplant is reported to increase the risk of TA-TMA (40). Similarly, in the autologous setting, tandem myeloablative HCT for patients with neuroblastoma (utilizing first cyclophosphamide and thiotepa, followed 6–10 weeks later with carboplatin, etoposide, melphalan) has been linked to high rates of TA-TMA (18).

The use of HLA-mismatched donors has been routinely associated with higher rates of TA-TMA (6, 16, 37, 40, 41), as has the use of peripheral blood stem cells (34). Blood group A and B antigens are expressed on endothelial cells and may serve as targets of attack in the setting of HCT from minor ABO-mismatched donors. Many reports suggest that minor ABO mismatch is a risk factor for TRM (32, 42), and this may at least partially driven by increased TA-TMA (40, 43, 44). The use of ABO-incompatible platelets was noted to be a risk factor for the development of SOS (45) though to date this question has not been carefully studied in TMA.

The use of myeloablative conditioning is usually associated with higher rates of TA-TMA compared to reduced intensity conditioning (RIC) regimens (40, 41, 46), however, which specific components of conditioning are most responsible remain to be worked out. Total body irradiation (TBI) has been linked to development of TA-TMA (16, 47), especially in patients with low body mass index (48). Once administered, fludarabine undergoes rapid dephosphorylation in the plasma to F-ara-A (9-beta-D- rabinofuranosyl-2-fluoroadenine). This molecule has been associated with endothelial injury and TA-TMA (49, 50). In adults undergoing non-myeloablative HCT, 16% of patients had high F-ara-A levels, which correlated with increased risk of transplant-related mortality (51). A pediatric analysis of F-ara-A levels did not show a correlation with TRM, though the rate of TRM in the studied patients was low, limiting power (52). The lack of anti-thymocyte globulin in the conditioning regimen is also associated with higher rates of TA-TMA. This is likely due to higher rates of GVHD in patients who do not receive ATG, as development of GVHD is linked to TA-TMA (discussed below).

In patients with neuroblastoma, early recovery of ANC is associated with increased risk of SOS (53). Although there is no data to suggest that the use of G-CSF to hasten neutrophil recovery post-HCT increases the risk of TA-TMA, given the role of NETs (discussed below) in exacerbating the endothelial injury, further investigation of whether there is a link between G-CSF and TA-TMA is warranted.

3. *Post-Transplant Event Risk Factors*

The use of certain medications post-HCT has been associated with increased risk of developing TA-TMA. The best-appreciated agents are the CNIs, cyclosporine and tacrolimus, especially when given in combination with sirolimus (37, 40, 41, 46, 54). The mechanism appears to be at least due to decreased levels of nitric oxide (NO) and vascular endothelial growth factor (VEGF). *In vitro* there is evidence that cyclosporine alone is worse than tacrolimus alone and similar to tacrolimus plus sirolimus (55), though a major difference has not been demonstrated clinically.

It is now well appreciated that acute GVHD is tightly linked to the development of TA-TMA (7, 16, 37, 40, 41, 46, 56). Elevations in ST2, a marker of endothelial injury, have been reported in corticosteroid-resistant GVHD (57), and this finding may link TA-TMA as a form of endothelial GVHD (58–61).

The development of certain infections post-HCT have been associated with increased TA-TMA. Although TMA itself and its treatment likely predisposes to the development of bacteremia, this pathway is bidirectional, for multiple episodes of bacteremia are strongly linked to subsequent development of TA-TMA (62). This is likely mediated by the release of neutrophil extracellular traps (NETs) into the vasculature (12, 63). NETs cause platelet aggregation, thrombin activation, and fibrin clot formation (64). Building upon these observations, since administration of G-CSF to healthy volunteers increases NET levels (65), one possible hypothesis is that G-CSF use in HCT recipients may predispose to or perpetuate TA-TMA, though to date there are no data analyzing this potential risk factor. Fungal infections, especially aspergillus, have been linked to TA-TMA (6, 16), possibly mediated via the same pathway by which the angioinvasion process leads to endothelial activation.

Double-stranded (DS) DNA viral infections such as CMV and HHV-6, especially when occurring together, have been liked to TA-TMA (16, 37, 56, 66). This process may be mediated via production of cytokines such as TNF-alpha and IL-1. High levels of BK viremia have also been linked to TA-TMA (67). The role of DS-DNA viral infection in propagating TA-TMA may explain why the use of alemtuzumab has not been associated with decreased risk (unlike has been seen with ATG), given the increased rates of infections noted with this agent (68).

Therefore, risk-assessment for TA-TMA has to occur at two time-points, pre-HCT based on intrinsic and transplant-related factors, but also post-HCT in the setting of new triggers such as GVHD or infections. Higher-risk patients would likely benefit from enhanced monitoring and clinicians having a higher index of suspicion for the diagnosis of TMA.

**Figure 1:**
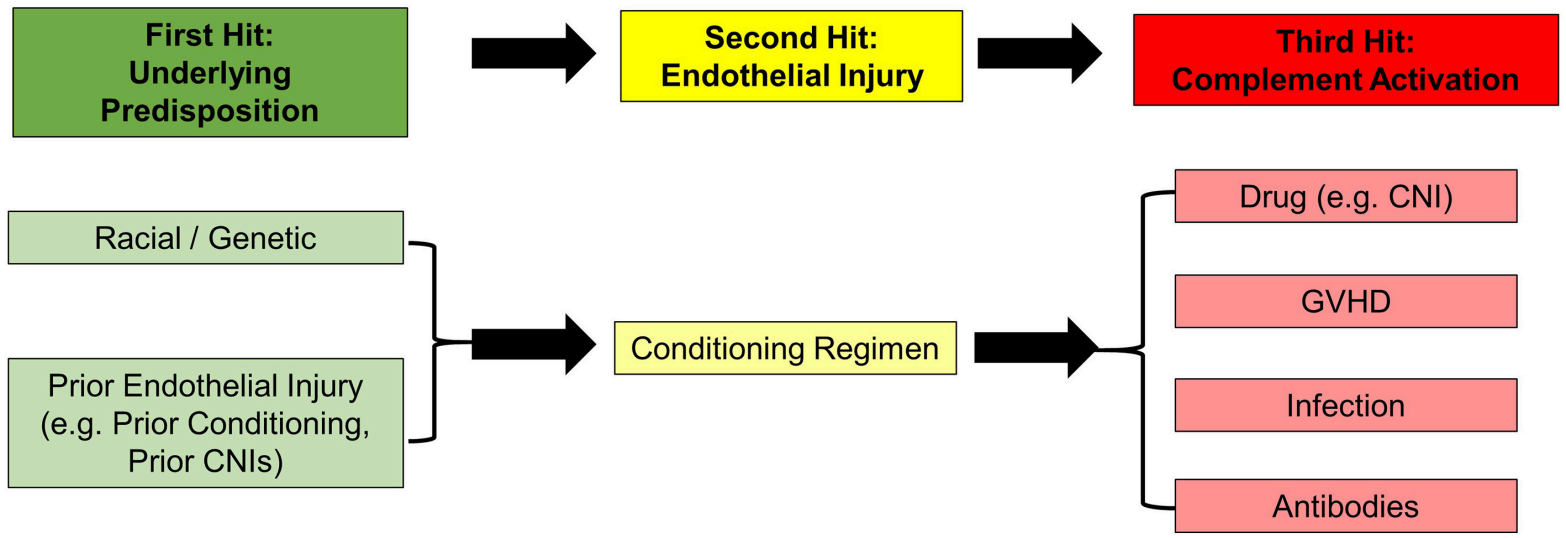
The “Three-Hit” hypothesis for the development of TA-TMA.

An analysis of the aforementioned risk factors for the development of TA-TMA lends support to a “*Three-Hit Hypothesis*” (Figure 1) (69). First, patients with either an underlying predisposition to complement activation (e.g., racial or genetic) or a pre-existing endothelial injury (e.g., prior myeloablative HCT or prolonged CNI use in SAA) (Hit 1), further endothelial injury is mediated by the delivery of a toxic conditioning regimen (Hit 2). Then, a third hit occurs which perpetuates continued endothelial injury and represents a threshold level for triggering activation of the complement system. This can be caused by medication toxicity (CNI, sirolimus), alloreactivity (GVHD), infectious (bacterial or viral), antibody-mediated, or some combination of all of these (Hit 3). As we learn more about the risk-factors for the development TA-TMA, this hypothesis may require further modification.

## Treatment of TA-TMA

Although definitive proof is lacking, by analogy to SOS, another endothelial damage syndrome, early therapy is predicted to result in improved survival and long-term outcomes, therefore close monitoring of at-risk patients is potentially very important. One simple measure to suggest patients at high-risk for doing poorly is the “TMA Index,” calculated by dividing the LDH (in IU/L) by the platelet count (in X10^9^/L) (16). Patients with a score of <20 typically do well, while those with a score of >100 have very poor outcomes. Proteinuria and an elevated sC5b9 level are also poor prognostic markers.

### Adjunctive Treatment

1. Anti-hypertensives: All patients with TA-TMA have hypertension and require multiple anti-hypertensives. However, not all agents may be equally beneficial. Because of the involvement of the renin-angiotensin system in mediating hypertension in TA_TMA, blocking this system with ACE-inhibitors or angiotensin 2 receptor agonists may be preferred (1, 70). However, in patients with significant kidney injury, calcium channel blockers may initially be safer due to their vasodilatory properties.
2. Erythropoietin and Thrombopoietin: Both PRBCs and platelet units contain plasma with significant amounts of complement (71, 72). Therefore, transfusions in patients undergoing complement blockade may be counter-productive and should be limited as much as possible (1), and volume-reduced platelets may be considered when transfusions are required. Patients with significant renal injury from TA-TMA may be erythropoietin (EPO) deficient, and careful administration of exogenous EPO to support reticulocytosis should be considered (73). Similarly, if immature platelet fractions are low, careful use of thrombopoietin receptor agonists, such as oral eltrombopag or subcutaneous romiplostim, may be considered, though there are currently no data in TA-TMA patients.

### Specific Treatment Algorithm

**Figure 2:**
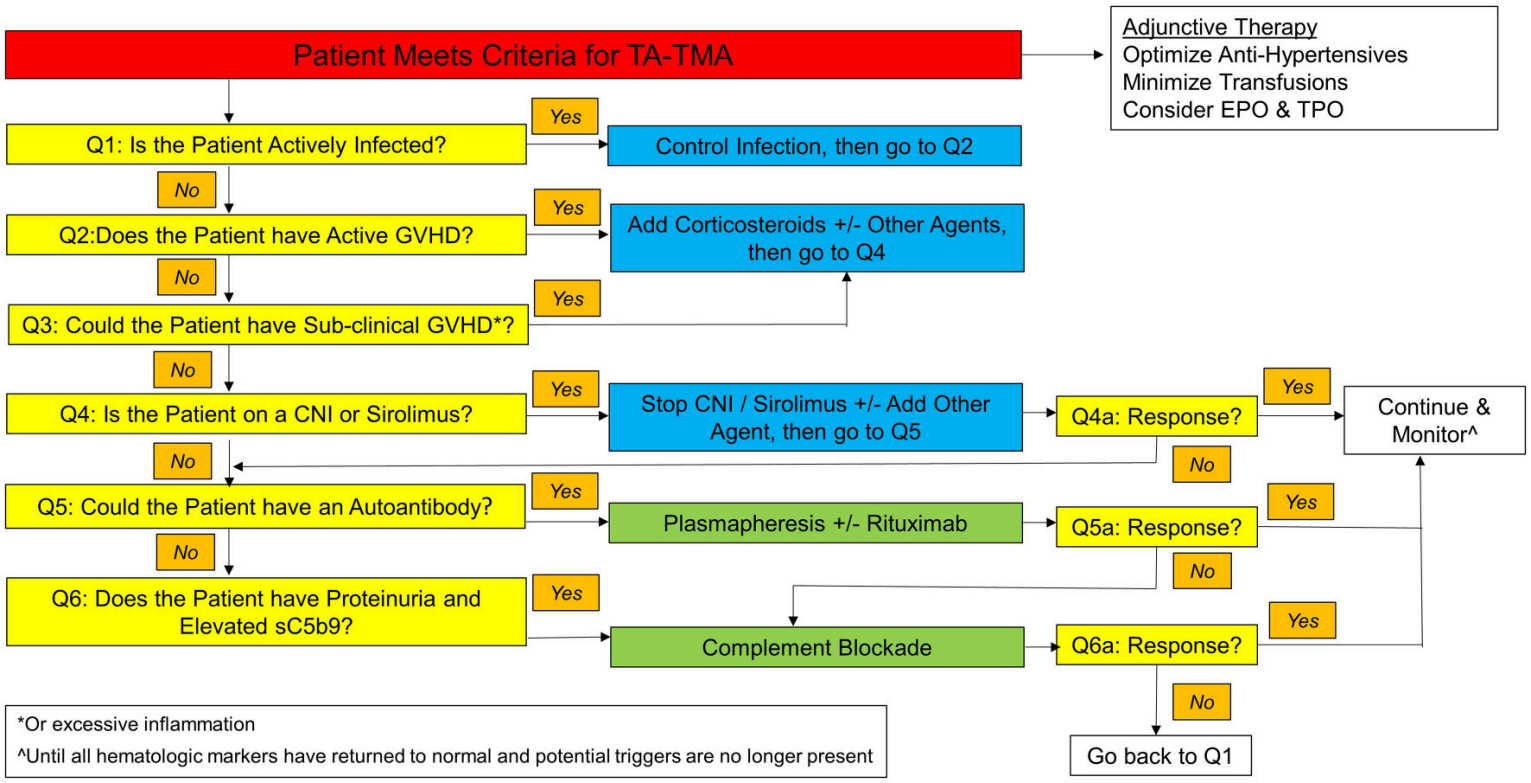
TA-TMA treatment algorithm.

Various therapies have been considered for TA-TMA, but overall survival rates remain worse than for the general population of post-HCT patients without TA-TMA (39, 41). An algorithm for the approach to treatment is proposed in Figure 2. Once a patient meets criteria for TA-TMA, it asks a series of questions to help guide the clinician to the treatment path most likely to improve the symptoms of TA-TMA and thereby optimize survival and long-term renal function. This algorithm contains several recommendations which are variably supported by studies (as noted), as well as anecdotal experience, and can be adapted to match an individual institution's unique situation. The first five questions relate to recognizing and treating the various triggers of TA-TMA, as it is unlikely to resolve until these triggers have also been brought under control.

Question 1: Is the patient actively infected?

Certain infections appear to contribute to the endothelial injury of TA-TMA. Anecdotally, this can include low-level viremia from DS-DNA viral infections. All efforts to suppress such viruses to the level of PCR-negativity should be employed, including modulation of immunosuppression, anti-viral agents, and potentially virus-specific cytotoxic T-cells (VS-CTLs). One report demonstrated that TA-TMA will not resolve if the underlying infection is not controlled (6). Unfortunately, many currently available anti-viral agents can further induce renal injury, and the potential benefit of modulation of immunosuppression and VS-CTLs needs to be carefully weighed against the risk of triggering alloreactivity.

Question 2: Does the patient have active GVHD?

There is clear evidence that active GVHD propagates the endothelial injury of TA-TMA. All efforts to minimize alloreactivity should be employed. The mainstay of this would be treatment with corticosteroids, though for patients without full response, consideration of other immunosuppressive agents should be considered, especially those which might target cytokine pathways also implicated in the TA-TMA pathway, such as TNF-a and IL-1 (22, 74, 75). One report demonstrated that TA-TMA will not resolve if the triggering GVHD is not controlled (6).

Question 3: Could the patient have sub-clinical GVHD (or excessive inflammation)?

Despite a lack of clinical GVHD, a patient may still have sub-clinical alloreactivity driving endothelial injury. We have anecdotally observed improved response to eculizumab in autologous recipients as compared to those undergoing allogeneic HCT, suggesting that unappreciated GVHD-mediated endothelial injury may exist in some patients. This can be difficult to document, but measurement of the CRP may provide information about the global state of inflammation. Another clue might be the state of donor T-cell chimerism. It has been shown that mixed T-cell chimerism implies a state of tolerance between donor and host, while full donor T-cell chimerism suggests that tolerance has been broken and the patient is at risk for (potentially sub-clinical) GVHD (76, 77). In this setting, or in autologous patients, careful use of corticosteroids may be considered in patients with other signs of excessive inflammation, such as engraftment syndrome.

Question 4: Is the patient on a CNI (or sirolimus)?

For patients who develop signs of TA-TMA while receiving CNIs for prophylaxis or treatment of GVHD, the first intervention is typically to rapidly discontinue the CNI (16). Depending on the clinical context, the patient may require the initiation of an alternative anti-GVHD agent. Typically, corticosteroids or mycophenolate mofetil is employed in these situations. Another class of agents used in this situation are IL-2 blockers (78), with basiliximab the currently available agent. Sirolimus is likely a poor option in this setting due to its association with TA-TMA when combined with tacrolimus. Discontinuing or changing the GVHD drug does not always reliably resolve TA-TMA; however, if caught early, this strategy may be sufficient for some patients (6, 7).

Question 5: Could the patient have an autoantibody?

One hallmark feature of TA-TMA, unlike TTP, is a general lack of response to therapeutic plasma exchange (TPE) (19, 44, 79, 80). This is likely because most patients do not have an autoantibody driving the pathophysiology of their TMA. However, in the subset of patients with an autoantibody against Factor H or other endothelial proteins, the best therapy may indeed be antibody removal via TPE, in combination with blockade of further production via administration of rituximab, given immediately after a TPE session (1, 31, 81).

TPE is invasive, as it often requires placement of a dedicated pheresis line, placement of which can be risky in a severely thrombocytopenic patient. If initiated, TPE should be performed daily at first (80). Other potential benefits of early initiation of TPE include: replacing dysfunctional complement regulators, removing in?ammatory cytokines, and removing promoters of endothelial damage, such as Ang-2, circulating endothelial cells, endothelial micro-particles, and circulating free hemoglobin (which binds to NO) released from damaged RBCs (10, 19, 82). We have anecdotally noted that LDH levels decrease in response to TPE faster than other hematologic parameters respond, which may be simply a matter of clearance but not actual disease resolution, so this lab marker alone should not be used to guide treatment course.

If a patient responds to TPE, it should be continued daily until complete resolution of the abnormal hematologic markers. TPE is then weaned to every other day for a period of about 2 weeks, then to twice weekly, and finally discontinued (80).

Question 6: Does the patient have proteinuria and/or an elevated sC5b9 level?

In pediatric patients, the best prognostic criteria identified to date has been the presence of proteinuria or an elevated sC5b9 (MAC complex) level (2). Patients with either abnormality should be strongly considered for treatment with terminal complement blockade, while patients with both should absolutely be started on treatment. Eculizumab is a monoclonal antibody against C5 that prevents formation of the MAC. In patients with elevated sC5B9 levels, use of eculizumab is associated with significantly improved overall survival compared to untreated controls (56 vs. 9%; *p* = 0.003) (79). Another report confirmed excellent initial responses to eculizumab, but cautioned that long-term overall survival remained poor in patients with TA-TMA (83).

While eculizumab levels can potentially be performed and correlate with response, practically, a fully suppressed CH50 is sufficient to demonstrate complete complement blockade (79). The optimal dosing to achieve this may be different than the package insert, as it has been demonstrated that twice-weekly doses are often initially needed (79). Eculizumab administration should not be combined with TPE, since the TPE clears the antibody itself and continually replenishes C5, thereby abrogating its effect. Furthermore, eculizumab may block function of rituximab, as the latter depends on the presence of complement (79). Therefore, the strategies of TPE ± rituximab and complement blockade are generally considered to be mutually exclusive (1).

In the US, eculizumab is only available through a Risk Evaluation and Mitigation Strategy (REMS). This entails counseling patients regarding the protective role of terminal complement from the development of meningococcal infections and providing them with a Patient Safety Card. Technically, patients are supposed to undergo meningococcal vaccination 2 weeks prior to initiation of eculizumab. However, this is not practical in the setting of TA-TMA, as patients may drastically worsen during those 2 weeks, but more importantly, early post-HCT, few patients will mount protective antibody titers to vaccines, so this could inadvertently leave patients open to risk. The standard practice in this setting is to provide antibacterial prophylaxis with agents such as penicillin or ciprofloxacin until eculizumab has been discontinued and CH50 levels return to normal (84).

For patients who do respond to eculizumab, a common dilemma is how long to continue the agent. If a pathogenic mutation in a complement gene is located (i.e., Primary aHUS), the answer is “lifelong.” However, most patients who develop TA-TMA do not have a significant mutation, but rather one or more minor gene variants. For these patients, withdrawal of complement blockade can typically be accomplished once all hematologic manifestations of the disease (e.g., thrombocytopenia, anemia, elevated LDH, elevated D-dimers, low haptoglobin) have resolved (1, 79). The median number of doses to accomplish this is 10, but with a wide range of 4–52 (85). Renal manifestations of the disease (e.g., hypertension, creatinine elevation, proteinuria) are less useful as markers of when to discontinue therapy, as they may remain permanently abnormal in the setting of significant renal injury (39).

Trials are currently underway to evaluate the use of ravulizumab-cwvz (formerly ALXN1210), a long-acting complement inhibitor, in pediatric patients with atypical HUS. The data for this agent are currently limited to patients with PNH, so its use in TA-TMA patients should be applied with caution (86).

The typical TA-TMA patient who discontinues complement blockade will not have recurrence of symptoms (79, 85). However, if patients continue to have potential triggers of endothelial injury or complement activation, then complement-blockade should generally be continued until that trigger is no longer present. For example, a patient with neuroblastoma who develops TA-TMA after a tandem HCT will then typically receive irradiation to their original tumor bed, an area that for most patients includes a portion of the kidney. It is unclear whether post-HCT immunotherapy with dinutuximab and IL-2 or GM-CSF is a sufficient trigger, but small numbers of patients have successfully gone through this treatment without eculizumab on board (18).

Questions 4–6A: Is the patient responding to the intervention?

As noted above, most patients with TA-TMA will not respond to plasmapheresis. One of the critical questions facing a physician caring for a patient with TA-TMA, however, is how long to wait for signs of response to an intervention before deciding it is a treatment failure. As a general rule of thumb, it takes at least 2–3 weeks to observe hematologic responses to therapy (1, 6, 80).

While complement blockade has significantly improved outcomes for patients with TA-TMA, a significant number (30–40%) of patients do not respond (79, 85). For patients who are not responding to complement-blockade, careful attention must be paid to other factors which might be perpetuating endothelial injury, with the recursive application of the questions in the algorithm in Figure 2. If all of these options have been exhausted, then alternative therapies must be considered.

### Alternative Therapies

- Other Complement Blockers: Eculizumab works by blocking the cleavage of C5 into C5a and C5b. However, C3b continues to build up on RBCs, which then can be cleared in the spleen, so extravascular hemolysis may continue (87). Furthermore, some C5 variants prevent eculizumab binding. Therefore, for patients with complement-driven disease, other complement inhibitors are being actively evaluated in trials, including coversin, a recombinant C5 inhibitor (88), and OMS721, a human monoclonal antibody that inhibits mannan-binding lectin-associated serine protease-2 (MASP-2).
- Soluble Thrombomodulin: TM is a glycoprotein on the surface of endothelial cells that, in addition to binding thrombin, regulates C3b inactivation by factor I. Antibodies against it have been linked to the development of aHUS. A small case series in Japan demonstrated a beneficial role for administration of recombinant TM to patients with TA-TMA (89). Unfortunately, this product is currently unavailable in the United States.
- Defibrotide: Defibrotide is a mixture of single-stranded oligonucleotides with endothelial protection properties mediated via inhibition of TNF-alpha and via its pro-fibrinolytic, anti-thrombotic, anti-inflammatory, and thrombolytic properties. It is FDA-approved for the treatment of SOS, another endothelial damage complication of HCT, and it was therefore hypothesized that it would play a role in the treatment of TA-TMA. Several reports suggest a possible benefit (16, 81, 90, 91). The biggest concern with using defibrotide in a patient with TA-TMA, who is typically profoundly thrombocytopenic, is bleeding, though the randomized trial in SOS prevention did not demonstrate a difference in bleeding events between the intervention and control arms (92). When used for management of SOS, standard practice is to keep the platelet count above 30 × 10^9^/L. This may be challenging, however, given the general desire to limit platelet transfusions (as noted above).

## Potential Approaches to TA-TMA Prophylaxis

None of the currently available treatments of TA-TMA have resulted in 100% response rates. With the hypothesis that it is easier to prevent a disease than treat it once fully established, there have been many efforts to identify a safe and effective prophylaxis regimen. While some of the TA-TMA risk factors are not easily changed, others may be amenable to modification. These approaches can be divided into those that improve baseline endothelial health, those that decrease the initial endothelial injury, and those that limit the “Third Hit.”

1. Improving baseline endothelial health
  - Vitamin D: In pediatric HCT patients, low baseline Vitamin D levels have been associated with worse overall survival (93, 94). *In vitro*, it has been demonstrated that Vitamin D supplementation can protect endothelial cells from radiation-induced damage (95). Although not yet studied, it is possible that a trial of Vitamin D normalization pre-HCT would partially abrogate TA-TMA.
  - Eicosapentaenoic acid (EPA): EPA, one of the active ingredients in fish oil, has been shown in one small trial to decrease TA-TMA, potentially via reduction of cytokines and stimulation of NO production (96). Larger trials are warranted.
2. Limiting/repairing endothelial injury from conditioning
  - Individualized-Dosing: As the initial injury to the endothelial cells is thought to be mediated by the damage caused by the conditioning regimen, we hypothesize that individualized dosing of these agents to account for patient-specific differences in metabolism could limit TA-TMA. An analogous prototype is busulfan, where high exposures are associated with development of SOS, GVHD, and TRM (97). PK-directed individualized dosing of cyclophosphamide and fludarabine already exist (52, 98), and studies are ongoing for melphalan, thiotepa, and clofarabine.
  - Allopurinol: Uric acid is a product of dying cells, and levels may become elevated following conditioning, especially in patients with high disease burdens coming into transplant. Elevations in uric acid have been shown to mediate endothelial injury, possibly via NET formation (99). A standard approach at some centers is to administer allopurinol through conditioning, and a small trial of rasburicase demonstrated lower rates of GVHD (100), though a formal trial of allopurinol to prevent TA-TMA has not been performed.
  - Defibrotide: As noted above, defibrotide may play a role in the treatment of TA-TMA. Defibrotide started the day prior to conditioning has been demonstrated to effectively provide prophylaxis against the development of SOS in high-risk patients (92). *In vitro*, defibrotide protects against endothelial injury induced by fludarabine and CNIs (50, 55). A trial of defibrotide prophylaxis in patients high-risk for development of TA-TMA is underway (NCT#03384693).
  - Statins: Statins have a potential preventative role in TA-TMA, as they have been shown to enhance endothelial cell function in HCT patients (101). One retrospective report in adults demonstrated significantly lower rates of TA-TMA when patients were given pravastatin (20 mg/day) starting on Day−1 (38). A pediatric trial has not been performed.
  - N-acetyl-L-cysteine (NAC): NAC is a synthetic anti-oxidant derived from cysteine that acts by removing reactive oxygen species (ROS) and regulating several functions of the immune system. NAC can act directly, by neutralizing ROS, or indirectly, by increasing glutathione biosynthesis. In a mouse model of shiga-toxin mediated aHUS, NAC significantly limited the endothelial injury mediated via ROS (102). A small case series suggested a benefit in patients with classic TTP via reduction of ultra-large VWF multimers (103), but data are currently limited in patients with TA-TMA.
3. Limiting the “Third-Hit”
  - CNI-free GVHD prophylaxis: Since CNIs are commonly implicated in the pathophysiology of TA-TMA, one hypothesis is that their elimination from GVHD prophylactic regimens should decrease the incidence of TA-TMA. Thus far, studies of *ex vivo* or *in vivo* T cell depletion in the matched donor setting have not reported on TA-TMA rates (104), though renal function at 1 year post-HCT seems improved (105). However, TA-TMA has been observed in both autologous transplants and *ex vivo* T-cell depleted haploidentical HCTs, highlighting that CNIs are not the sole trigger of endothelial injury.
  - Improved GVHD prophylaxis regimens: Since the endothelium appears to be a target of GVHD, optimized prophylaxis regimens that inhibit GVHD without increasing infections would theoretically significantly decrease rates of TA-TMA. Currently, the most promising agent in this regard is abatacept (106), though data on rates of TA-TMA after abatacept-containing regimens are still lacking.
  - Improved Antimicrobial Prophylaxis: Because both bacterial infections and DS-DNA viral infections are possible mediators of the “Third Hit” of post-HCT endothelial injury, interventions designed to minimize them may have a secondary effect of also lowering the incidence of TA-TMA. Levofloxacin has recently been shown to decrease bacteremia in pediatric HCT recipients (107). New anti-viral agents, such as letermovir and brincidofovir, may also help minimize reactivations that drive endothelial injury, though data on TA-TMA rates in patients receiving newer prophylactic agents are currently lacking.
  - Prophylactic Rituximab: As various endothelial-directed antibodies may play a role in triggering TA-TMA, prophylactic rituximab administration may abrogate this risk. Rituximab has been used in the peri-transplant period to prevent relapse, EBV reactivation, and GVHD, especially in *ex vivo* T-cell depleted HCTs (108, 109), but data on TA-TMA rates are currently lacking.

## Future Directions

The field of TA-TMA would benefit from several critical advances, including:

1. Development of a single diagnostic test with high sensitivity, high specificity, and fast-turn around. One of the major current limitations to rapid diagnosis (and hence treatment) is the current reliance on a complex algorithm devised of clinical and laboratory findings which may not all appear at the same time. Several potential tests have been proposed, including:
  - MAC complex: Soluble C5b9 levels at Day 28 have been tightly linked to later development of TA-TMA (110), however, given that elevated levels are also associated with poor outcomes, an earlier marker is probably required.
  - PAI-1: High levels of PAI-I have been associated with development of TA-TMA (22, 111). However, the utility of PAI-1 as a screening or diagnostic test has not been rigorously evaluated in a large, multi-center fashion.
  - Thrombomodulin: High levels at Day 14 post-HCT have been significantly linked to subsequent development of TA-TMA (111), and more recently, a panel of TM plus D-Dimers, ANG2 and CRP has been proposed (21). Unfortunately, a CLIA-approved test for TM does not yet exist in the US.
  - Calpain: Calpain is a calcium-dependent, non-lysosomal cysteine protease that functions as a platelet-aggregating factor. A small study showed a correlation with the presence and severity of TA-TMA, as well neurologic symptoms (112). Unfortunately, a CLIA-approved test for calpain does not yet exist in the US.
  - Haptoglobin degradation product: A novel biomarker of haptoglobin fragments (consumed during the hemolysis of TA-TMA) was recently identified, though it is still only available as a research test at this time (113).
2. Establishment of a robust risk-scoring system. Although a number of risk-factors for the development of TA-TMA have been identified, their relative (and potentially additive) roles have yet to be conclusively determined to enable robust delineation of patients at very low and very high risk for TA-TMA. Identification of a high-risk group would potentially enable enhanced screening, and if a safe, effective, and (ideally) inexpensive prophylactic agent were identified, this would facilitate trials in those highest-risk patients.
3. Further consideration of routine up-front genetic testing. Theoretically, HCT patients with *a priori* known variants in complement genes might be candidates for a trial of prophylactic complement blockade during the immediate post-HCT period when endothelial damage is at its peak.
4. Formal evaluation of the prophylactic strategies noted above with the best risk-to-benefit profile. The short list for such trials would include vitamin D, EPA, allopurinol, statins, and defibrotide.

## Conclusions

TA-TMA is the end result of a complex pathophysiologic injury to the endothelium in pre-disposed patients mediated by a combination of conditioning-induced injury and a “Second Hit” from medications (such as CNIs), GVHD, infections, or antibodies. In recent years, there has been an increased awareness of the risk of developing TA-TMA in pediatric HCT recipients. However, given the current diagnostic limitations, a high index of suspicion must be maintained, especially in high-risk patients during the highest-risk time period post-HCT (~21–100 days). This is similar to the careful attention that pediatric transplant physicians have historically paid to the development of SOS and acute GVHD, both of which may overlap with TA-TMA. The clinical TA-TMA triad of “*hypertension* (requiring one more antihypertensive than expected for the clinical situation), *thrombocytopenia* (esp. platelet transfusion refractoriness), and *elevated LDH*” should be constantly kept in mind. All institutions should strongly consider routinely monitoring LDH twice weekly for the first 100 days post-HCT, at least in high-risk patients. Once TA-TMA is diagnosed, a simple treatment algorithm can help guide the combination of interventions required to halt further progression of endothelial injury and allow the healing process to commence. In the future, one or more agents may potentially help improve baseline endothelial health and limit endothelial injury from both conditioning and the “Second Hit.”

## Author Contributions

All authors listed have made a substantial, direct and intellectual contribution to the work, and approved it for publication.

### Conflict of Interest Statement

CD has received consulting fees from Alexion, Inc. (manufacturers of eculizumab) and Jazz Pharmaceuticals (manufacturers of defibrotide). CH receives research funding from Jazz Pharmaceuticals. KS has received research funding from Alexion, Inc. The authors declare that the research was conducted in the absence of any commercial or financial relationships that could be construed as a potential conflict of interest.
